# Robustness of rigid and adaptive networks to species loss

**DOI:** 10.1371/journal.pone.0189086

**Published:** 2017-12-07

**Authors:** Savannah Nuwagaba, Feng Zhang, Cang Hui

**Affiliations:** 1 Centre for Invasion Biology, Department of Mathematical Sciences, Stellenbosch University, Matieland, South Africa; 2 MOE Key Laboratory for Grassland Ecosystem and Center for Quantitative Biology, Gansu Agricultural University, Lanzhou, China; 3 Mathematical and Physical Biosciences, African Institute for Mathematical Sciences, Muizenberg, South Africa; Universidad Rey Juan Carlos, SPAIN

## Abstract

Controversies in the complexity-stability debate have been attributed to the methodologies used such as topological vs. dynamical approaches or rigid vs. adaptive foraging behaviour of species. Here, we use a bipartite network model that incorporates both topological and population dynamics to investigate the robustness of 60 real ecological networks to the loss of generalist and specialist species. We compare the response in both adaptive and rigid networks. Our results show that the removal of generalists leads to the most secondary extinctions, implying that conservation strategies should aim to protect generalist species in the ecosystem. We also show that adaptive behaviour renders networks vulnerable to species loss at initial stages but enhances long term stability of the system. However, whether adaptive networks are more robust to species loss than rigid ones depends on the structure of the network. Specifically, adaptive networks with modularity < 0.3 are more robust than rigid networks of the same modularity. Interestingly, the more modular a network is, the less robust it is to external perturbations.

## Introduction

Human activities are continuously driving species extinction in many ecosystems, threatening their function and the provision of ecosystem services [1–5]. Understanding the stability of ecological networks, i.e. how the systems respond to perturbation through both natural and anthropogenic means, has remained at the forefront of attention in ecological studies [3, 6–10]. Traditionally, the stability of a complex dynamical system can be mathematically determined by analysing the local and asymptotic behaviours of its trajectories [11–14]. However, such Lyapunov stability analysis only reflects one facet of how systems respond to perturbations, and can become clumsy when the dimension and complexity of the system reaches certain levels, which is often the case for ecological networks [15]. Alternative methods have been designed that cater for other facets of network stability, especially for large and complex systems where the traditional methodology fails [16–17]. To this end, robustness has been proposed to capture how ecological networks respond to the loss of species (nodes in the network). Although there are a variety of definitions of robustness in literature, all have quantified it as a measure of subsequent secondary extinctions due to species removal in an ecological network. In particular, robustness is defined as the fraction of species that need to be removed to result in a greater than 50% total loss of species in a food web [18]. A central goal for biological conservation is, thus, to identify networks with high robustness and potentially preserve processes that can enhance ecosystem robustness.

Robustness analysis is traditionally carried out in a topological test: nodes in a network are sequentially removed, and after each removal nodes left with no links are considered secondarily lost; the sequence of node removal is often considered as a function of node degree (high node degrees represent generalists, while low node degree represents specialists) [6,7,18]. For food webs’ response to the perturbation of species removal we have known[18]: (i) the removal of generalist species can cause more secondary extinctions than the removal of specialists; (ii) robustness increases with network connectance (the number of realised links divided by the number of possible links when fully connected—although connectance alone cannot determine robustness); (iii) the removal of some species (defined as functionally important) can lead to fatal consequences in the network. For the sequential removal of generalist species (i.e. highly connected species), to attain high robustness, a network needs to have high connectance, relatively uniform degree distribution and good expansibility (the absence of structural bottlenecks in a food web, whose removal separates the network into large isolated clusters) [7].

Although these studies have portrayed a clear picture of particular network architecture that can foster robustness, the static nature of the network topology (i.e. fixed binary interactions in a rigid network) is unrealistic [3, 4, 19]. Species often switch their interacting partners as a response to changing availability of habitats and resources [20–25]. The adaptive interaction switching can be further explained by the adaptive diet choice according to optimal foraging theory in varying environments where a predator will only forage a subset of potential preys to maximise the energy intake rate [26–28]. Earlier studies proved that adaptive behaviours can potentially favour stability particularly in antagonistic networks and food webs [10, 29–31]. Adaptive interaction switching can further affect species abundance and thus interaction strengths in ecological networks [32–33]. However, recently, Gilljam and colleagues showed that this switching behaviour could only be advantageous to individual consumers in a short term but harmful for the long-term network persistence because rewiring can result in resource overexploitation [34].

Here, we allow species to switch their interacting partners adaptively in response to the loss of species in ecological networks. The rule of adaptive interaction switching is designed to follow Russell Wallace’s definition of natural selection via the elimination of the unfit and random drift. The former makes species eliminate the worst link for them (i.e. selection or optimization), and the latter allows species to randomly find new link (i.e. explore other unlinked resources). The hybrid switching rule might imitate the real behaviour of species and has been shown to account for the majority of variation in observed network structures (e.g. node degree, nestedness and modularity) [32, 35]. Here, we plan to examine: (i) which network structures strongly affect network robustness; (ii) how the sequence of species removal affects network robustness; (iii) the effect of species’ adaptive behaviour on network robustness, and (iv) the impact of different levels of robustness threshold on our results. Importantly, for the first time, the robustness of rigid and adaptive networks is compared.

## Material and methods

Let us consider an antagonistic plant-herbivore network (host-parasite network in the same way), consisting of *m* plant species and *n* herbivore species. The population dynamics of plant *i* is controlled by its own density-dependent recruitment minus the loss due to feeding by herbivores, whereas the population dynamics of herbivore *j* is governed by the population increase rate due to foraging (depicted by Holling’s type II functional response) minus its mortality. This yields the following Lotka-Volterra model:

$$\begin{array}{l} \frac{1}{P_{i}}\frac{dP_{i}}{dt}=r_{i}-c_{i}P_{i}-\sum_{j}\frac{a_{ij}v_{ij}H_{j}}{1+h\sum_{k}a_{kj}v_{kj}P_{k}}\\ \frac{1}{H_{j}}\frac{dH_{j}}{dt}=-d_{j}+\sum_{i}\frac{b_{ji}a_{ij}v_{ij}P_{j}}{1+h\sum_{k}a_{kj}v_{kj}P_{k}} \end{array}$$ (1)

where *P*_*i*_ and *H*_*j*_ are the population sizes of plant *i* and herbivore *j*, respectively; *r*_*i*_ and *c*_*i*_ the intrinsic growth rate and the density-dependent coefficient of plant *i*; *d*_*j*_ the mortality rate of herbivore *j*. The last term depicts the functional response of the plants to the foraging by herbivores. Specifically, the binary diet matrix <*a*_*ij*_> indicates whether plant *i* is part of herbivore *j*’s diet (*a*_*ij*_ = 1) or not (*a*_*ij*_ = 0); the preference matrix <*v*_*ij*_> depicts the probability of herbivore *j* determining to feed on species *i* once met; the benefit matrix <*b*_*ji*_> represents the benefit received by herbivore *j* from consuming plant *i*; *h* denotes a herbivore’s handling time spent on a plant and is assumed to be equal for all species [36–37] (*h* = 0.1). Direct competition within the same trophic level is ignored as its impact on population dynamics is often much weaker than cross-trophic antagonistic interactions [9, 38–42]. We here emphasize indirect resource competition mediated by ecological network.

Due to lack of information from realistic networks, the values of initial population sizes were randomly assigned between 0 and 1. The values of intrinsic growth rates, density-dependent coefficients and the entries of the preference matrix, the entries of the benefit matrix were randomly assigned such as to ensure the persistence of all species in the network at equilibrium [43]. The assignment of different values to parameters does not affect the results [32]. The entries of the diet matrix were initially randomly assigned to be either 0 or 1, with the number of species and interactions being equal to the observation from 60 real networks (see S1 Table) and also ensuring no isolated species in the network. This diet matrix was then updated at each time step when numerically solving the model according to the following rule of interaction switch. During each time step, we first randomly select two herbivores: one drops from its diet the plant species that contributes the least to its fitness (i.e. per capita growth rate, *b*_*ji*_*a*_*ij*_*v*_*ij*_*P*_*j*_), and the other randomly add a new plant into its diet with a preference value randomly assigned between 0 and 1 (all the other parameters unchanged) [32].

**Fig 1:**
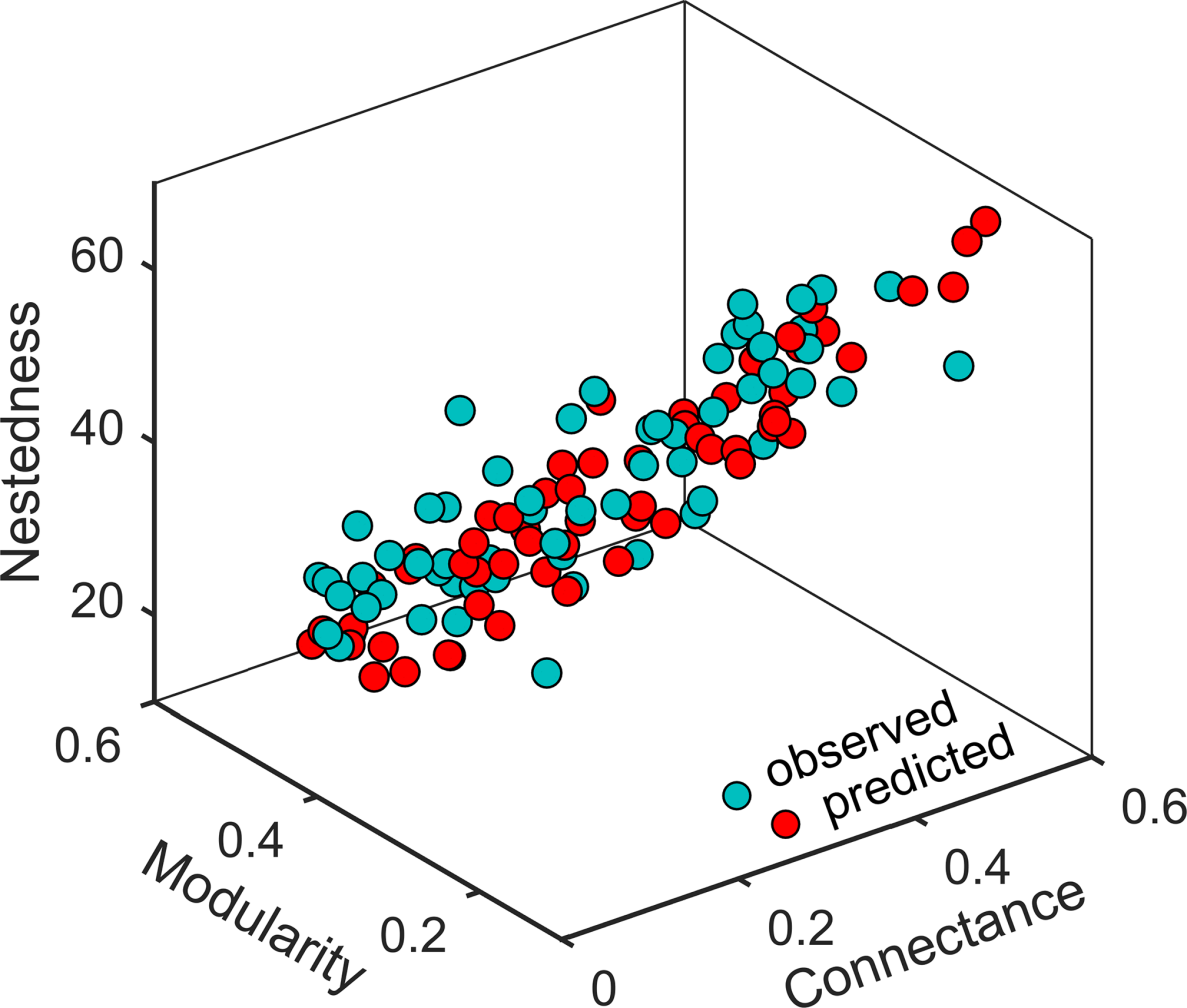
Predicted vs. observed network architecture. For each network, simulations were run to equilibrium and its architecture (modularity, nestedness, and connectance) recorded. Predictions are the averages of the last 200 time units after the dynamics stabilize.

We ran the model with an interaction matrix (i.e. the diet matrix) randomly assigned initially with an equal number of interactions as the observed networks in our previous work [32]. Each simulation corresponds to a specific real network. We tracked the interaction matrices, their modularity, nestedness and skewness of node degree distribution over time, with each time unit equalling *n*+*m* steps of interaction switching. Modularity was calculated by using the software NETCARTO based on simulated annealing [44] as the modularity optimisation technique [45–46] while nestedness was measured based on the overlap and decreasing fill (NODF) using the software ANINHADO 3.0 [47]. Before the removal of species, the model was run up to the time t = 150 to allow the network architecture to reach its equilibrium (normally when t = 20). An illustration of the performance of the model for predicting the structures of these 60 real networks was shown in Fig 1. The network was then subjected to a sequential removal of plant species.

For the sequential removal of generalist (specialist) species, a plant species which had the highest (lowest) number of interactions was removed from the network. The network was then allowed some time to reorganise via adaptive interaction switching. The time allowed for species to reorganise was proportional to the diversity of the network at the moment in order for each species to have a chance to respond to the change in the network. A herbivore was declared extinct if it had no interacting partner, while plant species were not allowed to go extinct even without interacting partners. The network obtained after each “adaptation period” was considered to be ready for the next species removal.

Robustness was mainly measured as the proportion of plant species that needed to be removed before at least 50% of the herbivore species went extinct (denoted by R50). This definition was modified here specifically for bipartite networks and slightly different from the one for food webs proposed before [18]. Other levels of robustness threshold were used to reflect the proportion of plant species that needed to be removed before at least a certain percentage of the herbivore species went extinct, in particular, R10, R30 and R70. To determine which variable contributes most to the level of robustness, we carried out the principal component analysis to group highly correlated variables. From each group one variable was then used in the generalised additive model fitting of robustness on these selected variables, with the importance of these variables in determining network robustness assessed. To compare with the traditional definition of robustness for rigid networks, all analyses were also run for networks without allowing for adaptive interaction switching.

## Results

**Fig 2:**
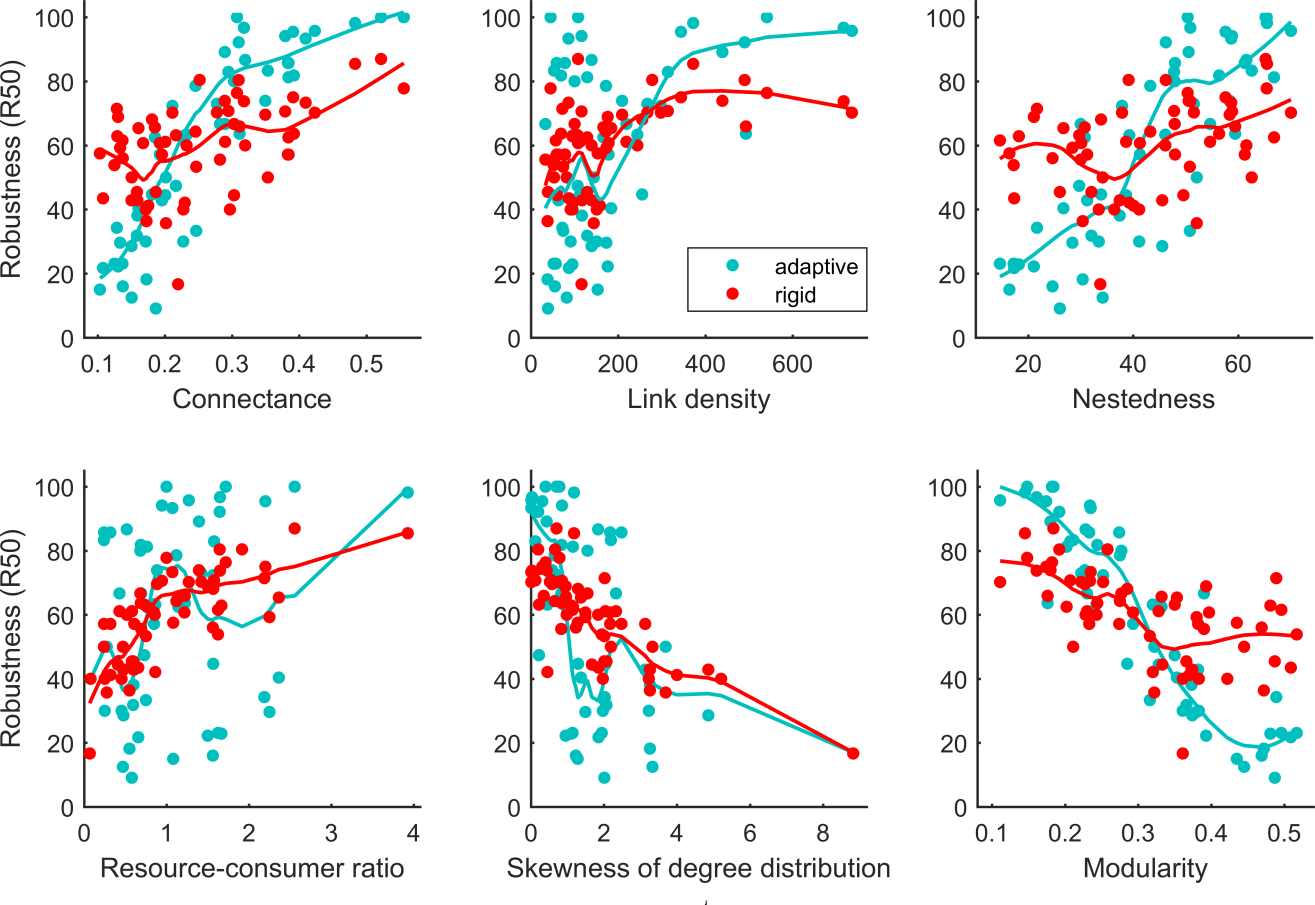
Robustness vs. network structure. For each simulation, the model was ran up t = 150, after which its structure was recorded. The generalist species were sequentially removed and the level of robustness recorded. Panels show the relationship between robustness and connectance, link density, nestedness, resource-consumer ratio, skewness of the degree distribution and modularity in both adaptive and rigid networks. R50 was used as the measure of robustness. Cyan lines indicate regressions for adaptive networks (cyan dots) while red lines for rigid networks (red dots). Spearman’s rank correlation coefficients are summarised in Table 1.

Robustness (R50) was significantly correlated with many network properties, including resource-consumer ratio, link density (number of interactions/links), connectance, nestedness, modularity, and the skewness and kurtosis of the node degree distribution (Table 1 and S1 Table) for both adaptive (cyan lines and dots in Fig 2) and rigid (red lines and dots in Fig 2) networks. In contrast, the correlation between robustness and species richness (*n*+*m*) was not significant (Table 1). The correlation between robustness and network architecture (nestedness, modularity and connectance) was stronger when species were allowed to switch than when they were assumed to be fixed as to who they interact with (Fig 2), while the opposite was true for other network structures (Table 1).

**Table 1:** Spearman’s rank correlations between network structure and robustness (R50, is the percentage of generalist resources that need to be removed before at least 50% of consumer species go extinct).

Variable	Adaptive network	Rigid network
RC ratio	0.264	0.764
Link density	0.389	0.508
n+m	-0.022	0.204
n×m	0.019	0.332
Connectance	0.872	0.453
Nestedness	0.807	0.364
Modularity	-0.899	-0.631
Skewness	-0.577	-0.767
Kurtosis	-0.480	-0.721

**Note:** RC ratio stands for resource-consumer ratio; the level of nestedness is measured by NODF; Skewness and Kurtosis are measured for the node degree distribution. Underlined correlations are not statistically significant (p > 0.05).

**Fig 3:**
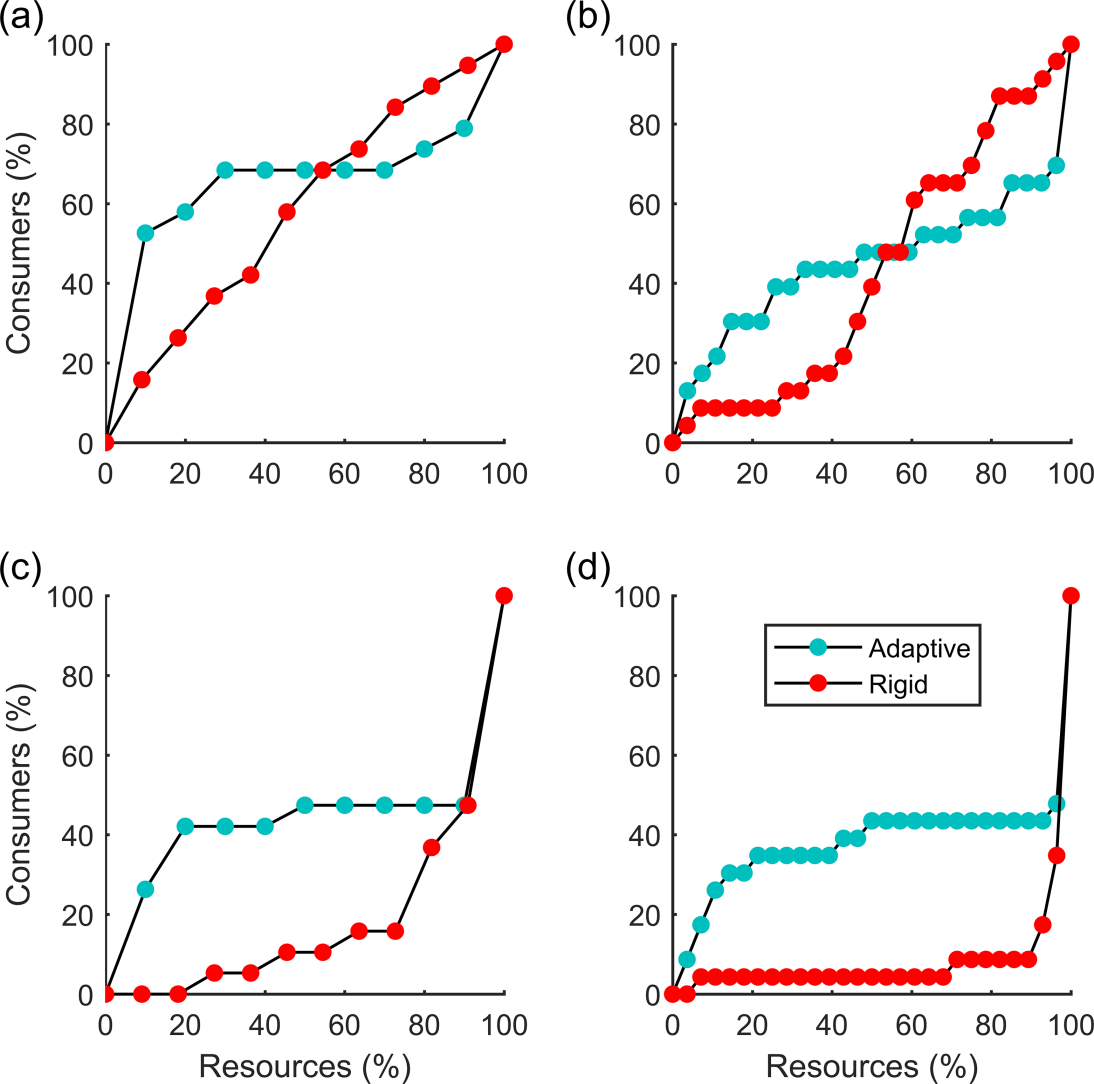
Robustness to the removal of species in adaptive and rigid networks. Panels (a) and (c) correspond to the real network, N41 while (b) and (d) correspond to N43 in S1 Table in the supporting information. For panels (a) and (b), generalist species were sequentially removed while for (c) and (d), specialists were sequentially removed. Points show the percentage of consumer extinctions that resulted from the removal of a certain percentage of generalist (a and b) or specialist (c and d) resource species from an adaptive or rigid network.

Although rigid networks were usually more robust than adaptive ones at the beginning of the removal of generalist resource species, they became less robust in the long run (Fig 3). Whenever the rigid network was more fragile to species removal compared to the adaptive one at the beginning, it remained less robust till the end (see Fig 3A and 3B). In cases where the adaptive network was less robust at the beginning to species removal, after a certain threshold (which was mostly never reached in specialist removals; Fig 3C and 3D), it later always became more robust than the rigid one, suggesting that species adaptive behaviour enhances robustness although it might initially enhance the fragility of networks.

**Fig 4:**
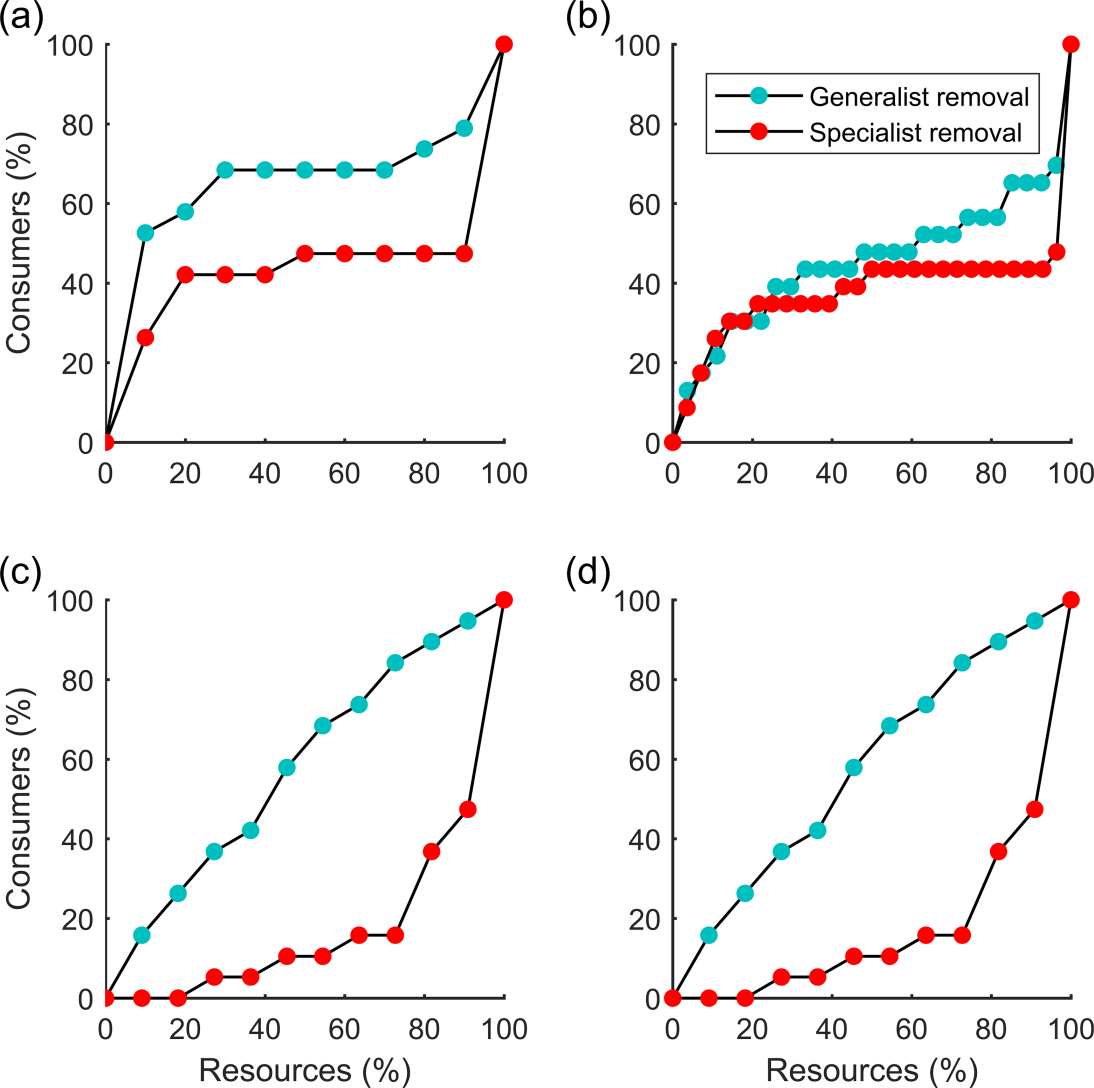
Robustness to the removal of generalists and specialists in two networks (N41 and N43 in S1 Table). Points show the percentage of consumer extinctions that result from the removal of a certain percentage of resource generalists and specialists. Panels (a) and (b) correspond to adaptive networks while (c) and (d) correspond to rigid networks.

**Fig 5:**
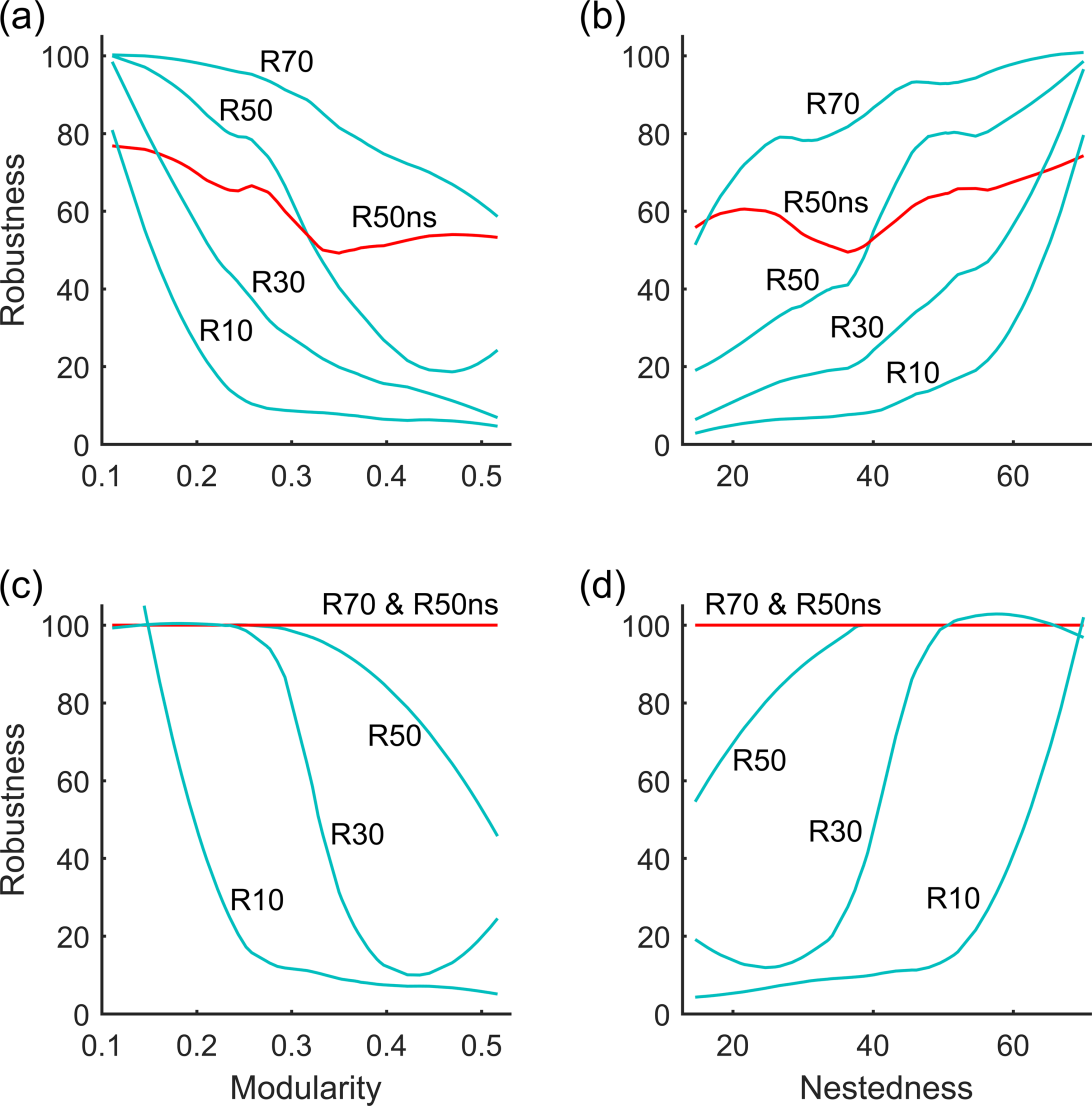
Genaralised additive model lines of fit of robustness with different threshold percentages on modularity and nestedness. R10—R70 correspond to the percentage of resources that need to be removed before at least 10–70% of consumer species go extinct in an adaptive network while R50ns corresponds to the percentage of resources that need to be removed before at least 50% of consumer species go extinct in a rigid network. Panels (a) and (b) indicate the removal of generalists while (c) and (d) indicate the removal of specialists.

Networks were more robust to the removal of specialists than to the removal of generalists whether using rigid or adaptive networks (Fig 4). However, while using the threshold percentage of consumers that go extinct after the removal of resources, we found that there were variations as to whether adaptive networks are more robust than rigid ones (Fig 5). That is, whether networks are more robust when rigid or adaptive depends on the threshold level used in the definition of robustness. For example, for the network in Fig 3B, if R30 is taken, the adaptive network (cyan dots) will be less robust than the rigid one (red dots), but if R70 is taken, the opposite will be true. For different levels of robustness threshold the impact of network structure (depicted by modularity and nestedness) on robustness can be different, with opposite signs of the regression slopes (Fig 4).

Importantly, the conclusion as to whether adaptive networks are more robust than rigid ones depends on the network structure. For instance, for networks whose modularity is less than 0.3 adaptive networks are more robust than rigid ones, and the robustness is more sensitive to changes in the structure of adaptive networks rather than rigid networks (comparing the steepness of the regression lines of R50 vs. R50ns in Fig 5). Overall, adaptive networks are more robust than rigid networks for weakly compartmentalized networks (modularity <0.3) and highly nested networks (NODF > 50) (Fig 5).

The principal component grouped variables of network structures that were used for explaining the level of robustness, with particular variables highly aligned with the principal component vectors. The three groups depict, respectively, network structure (including modularity, nestedness and connectance), network complexity (including species richness and link density) and network asymmetry (skewness and kurtosis of the node degree distribution and resource-consumer ratio) (S2 Table). The impact of network structure and complexity were under-rated in rigid networks while network asymmetry was emphasized, compared to the case in adaptive networks (Table 2). When selecting one variable from each group (in particular, modularity, link density and skewness) to explain robustness, modularity contributed the most to the level of robustness as dropping it from the generalised additive model will drastically reduce the variance explained (Table 2).

**Table 2:** Variance explained measured by adjusted R^2^ from the generalised additive model fitting of robustness on specific models.

Model	Adaptive network	Rigid network
Modularity + Link density + Skewness	0.854	0.712
Link density + Skewness	0.355	0.648
Modularity + Skewness	0.838	0.698
Modularity + Link density	0.839	0.454

## Discussion

Optimal foraging theory has been supported for being able to capture the realistic decision making of consumers. It suggests that consumers do not necessarily target all available resources but rather target only those that can maximise the energy intake rate [26]. However, because models based solely on optimisation of energy intake rate often exaggerate the structure of ecological networks, we have proposed the model where the optimisation process of adaptation was counterbalanced by the random drift [32], reflecting both profit seeking and risk aversion behaviour of consumers [28]. As the model can predict extremely well the observed structures of empirical networks of both plant-herbivore and host-parasite interactions, with 90% variance explained [32], implementing the same model for probing the relationship between network robustness and structure could have captured the essence of the stability in bipartite antagonistic networks amidst the loss of species.

Thierry et al. (2011) showed that the interaction switch as a species rewiring mechanism could increase the robustness especially in networks of low connectance [19]. However, Gilljamand colleagues [34] stipulate that adaptive behaviour of switching is a two-edged sword. It may be advantageous for individual consumers but harmful to the network as a whole. Adaptive networks were more robust than rigid networks because species under stress from losing interacting partners could switch and transfer their stress to other species but only until a certain threshold. Our results show that adaptive switch can enhance network robustness when the network is close to total collapse (i.e. species removal has led to most species being lost from secondary extinctions [a large threshold for the robustness measure]). For example, if we consider R70 as the measure of robustness, adaptive networks are generally more robust than rigid ones (Fig 3). This shows that, when only a few resource species remain (one or two in our simulations), adaptive switch enables consumers to coexist and persist with much flexibility, unlike the scenario in rigid networks. The case is only representative in communities at the verge of complete collapse, and thus allowing species the flexibility to adaptively switch to accessible resources could be important to ensure conservation success of stressed communities. Our simulations also suggest that adaptive networks can have higher secondary extinction at the beginning phase of species loss (Fig 3), which could serve as a warning sign to conservation management. This also implies that ignoring adaptive behaviours may often overestimate the stability of ecological networks.

Although it was realised that increased human disturbance generally led to the loss of poorly connected species [4], many studies have shown that their loss does not induce as many secondary extinctions as those induced by the loss of generalists species [4,6,48–49]. The loss of these poorly connected species or generalists may occur in the process of selective harvesting of specific species, which is common in human activities such as in the fishing industry [50]. Our results agreed that the consequence of species removal was much higher when generalists were removed than when were specialists (Fig 4), consistent with the previous studies [4, 6, 48–49]. With the increase in targeted ‘attacks’ in ecosystems, management strategies should be designed to prioritize the protection of generalist species more as they are critical to the stability of ecosystems [48].

Our results further demonstrated the crucial role of network structure in determining the level of robustness (Tables 1 and 2). Many studies have argued that a compartmentalized network may contain the effects of any disturbances (for example species loss) and hence enhance stability [8, 11, 51]. We showed that the more compartmentalized a network is, the less robust it will be to species removal (Table 1), contrary to many recent studies [8–9]. Previous studies have demonstrated that networks whose degree distributions are uniform are more robust to species loss [7, 18]. Highly compartmentalized networks are also highly skewed (Fig 2E and 2F), which could be one reason for reduced robustness in these networks [18]. Importantly, the measure of stability in these two papers [8–9] is not robustness but *persistence*, which is defined as the proportion of species that remain in a system at equilibrium. In contrast, we used robustness as the proxy of network stability and also allowed the system to first reach its equilibrium before introducing disturbances (species loss). It is possible that persistence and robustness measure different facets of network stability, potentially how networks combat against internal and external disturbances, respectively. In other words, the more compartmentalised a network is, the less robust it is to external disturbance, but more persistence to internal disturbance. Overall, although network robustness can be affected by a number of factors, network structure, in particular modularity strongly correlated with nestedness and connectance (Fig 1), plays the most important role in determining the level of robustness. The conclusion as to whether adaptive networks are more robust than rigid ones can potentially change, depending on the level of compartmentalization.

By allowing species to adaptively respond to changes in their environment, we demonstrated that biodiversity loss can affect a larger number of other species than expected. In fact, the consumer which switches to a new resource as a result of losing its own can turn into a native invader leading to overexploitation of the remaining resources [34]. Therefore, if we assume that the ecosystem consists of isolated species and that any species loss does not affect others, we are likely to underestimate the magnitude of the consequences of biodiversity loss especially during the early phase of disturbances. The early removal of species following any of the removal sequences (generalist or specialists) in our model resulted in more secondary extinctions when species were allowed to switch to new diets than when they were not allowed to. Although this was unexpected, it points to the fact that there is a need for a more inclusive measure of robustness or stability in order for us to make robust conclusions as to whether complexity begets stability in ecological networks. Meanwhile, we must value our knowledge of possible adaptive processes as they may have important implications for network robustness thus biodiversity maintenance and ecosystem function.

## Supporting information

S1 Fig Robustness vs. modularity and nestedness. (DOCX)

S1 Table A summary of results from all the simulations. (XLS)

S2 Table Principal component analysis of associated variables. (DOCX)

S3 Table Correlations between different levels of robustness to the removal of generalist species and variables of network structure. (DOCX)

S4 Table Correlations between different levels of robustness to the removal of specialist species and each of nine variables that describe of network structure. (DOCX)

S5 Table Variance explained measured by adjusted R^2^ from the generalised additive model fitting of robustness on specific models for different levels of robustness. (DOCX)

S6 Table Pearson’s correlation coefficients for different variables. (DOCX)
