# Supplementary material for: Robustness of rigid and adaptive networks to species loss
Source: PLoS One. 2017 Dec 7;12(12):e0189086. doi: 10.1371/journal.pone.0189086 (PMC5720727; doi:10.1371/journal.pone.0189086)
Supplement: S1 Fig — (DOCX) [file pone.0189086.s001.docx]

**S1 Fig: Robustness vs. modularity and nestedness.** For each simulation, the model was ran up t=150, after which its structure was recorded. Error bars indicate the standard deviation. The generalist species were sequentially removed in the adaptive networks and the level of robustness recorded. Networks used are N10, N22, N25, N38 and N60 in S1 Table. Although few networks were used, we were able to capture the relationship between robustness and network architecture (modularity and nestedness averaged for 30 replicates of each network) for different levels of robustness threshold (R10 - R70).
