# Supplementary material for: Robustness of rigid and adaptive networks to species loss
Source: PLoS One. 2017 Dec 7;12(12):e0189086. doi: 10.1371/journal.pone.0189086 (PMC5720727; doi:10.1371/journal.pone.0189086)
Supplement: S2 Table — (DOCX) [file pone.0189086.s003.docx]

**S2 Table:** **Principal component analysis of associated variables.**

| Variable | Factor Loadings (Marked loadings >0.63) | | |
| --- | --- | --- | --- |
|  | Factor 1 | Factor 2 | Factor 3 |
| RC ratio | -0.004978 | -0.392571 | 0.633656 |
| Link density | -0.380939 | -0.856420 | 0.230102 |
| n+m | 0.105016 | -0.971770 | -0.139808 |
| n×m | 0.047801 | -0.953779 | 0.208620 |
| connectance | -0.938927 | 0.145167 | 0.140079 |
| NODF | -0.977139 | 0.008308 | 0.005718 |
| MOD | 0.913157 | 0.296330 | -0.192535 |
| skewness | 0.220313 | 0.093422 | -0.940741 |
| kurtosis | 0.082434 | -0.095994 | -0.950608 |
| Prp.Totl | 0.320447 | 0.318722 | 0.262545 |
