# Supplementary material for: Robustness of rigid and adaptive networks to species loss
Source: PLoS One. 2017 Dec 7;12(12):e0189086. doi: 10.1371/journal.pone.0189086 (PMC5720727; doi:10.1371/journal.pone.0189086)
Supplement: S3 Table — (DOCX) [file pone.0189086.s004.docx]

**S3 Table:** **Correlations between different levels of robustness to the removal of generalist species and variables of network structure.**

| *Variable* | Marked Spearman’s rank correlations are significant at p < .05  N=60 (Casewise deletion of missing data) | | | | | | | |
| --- | --- | --- | --- | --- | --- | --- | --- | --- |
|  | R10gn | R30gn | R50gn | R70gn | R10gs | R30gs | R50gs | R70gs |
| RC ratio | 0.511 | 0.750 | 0.764 | 0.747 | 0.062 | 0.186 | 0.264 | 0.232 |
| Link density | 0.430 | 0.519 | 0.508 | 0.374 | 0.214 | 0.400 | 0.389 | 0.318 |
| n+m | 0.208 | 0.208 | 0.204 | 0.085 | -0.042 | -0.006 | -0.022 | -0.074 |
| n×m | 0.226 | 0.307 | 0.332 | 0.203 | -0.124 | 0.016 | 0.019 | -0.017 |
| connectance | 0.553 | 0.485 | 0.454 | 0.378 | 0.857 | 0.894 | 0.872 | 0.796 |
| NODF | 0.373 | 0.352 | 0.364 | 0.250 | 0.744 | 0.827 | 0.807 | 0.732 |
| MOD | -0.627 | -0.640 | -0.631 | -0.490 | -0.749 | -0.902 | -0.899 | -0.800 |
| skewness | -0.685 | -0.777 | -0.767 | -0.713 | -0.398 | -0.591 | -0.577 | -0.560 |
| kurtosis | -0.598 | -0.757 | -0.721 | -0.727 | -0.289 | -0.460 | -0.480 | -0.465 |
