# Supplementary material for: Robustness of rigid and adaptive networks to species loss
Source: PLoS One. 2017 Dec 7;12(12):e0189086. doi: 10.1371/journal.pone.0189086 (PMC5720727; doi:10.1371/journal.pone.0189086)
Supplement: S4 Table — (DOCX) [file pone.0189086.s005.docx]

**S4 Table:** **Correlations between different levels of robustness to the removal of specialist species and each of nine variables that describe of network structure.**

| Variable | Marked Spearman’s rank correlations are significant at p < .05  N=60 (Casewise deletion of missing data) | | | | | | |
| --- | --- | --- | --- | --- | --- | --- | --- |
|  | R10sn | R30sn | R50sn | R70sn | R10ss | R30ss | R50ss |
| RC ratio | 0.402 | 0.357 | -0.150 | -0.122 | 0.102 | 0.355 | 0.338 |
| Link density | 0.773 | 0.613 | 0.258 | 0.088 | 0.280 | 0.530 | 0.773 |
| n+m | 0.513 | 0.292 | 0.057 | -0.048 | -0.037 | 0.138 | 0.513 |
| n×m | 0.505 | 0.354 | 0.002 | -0.069 | -0.097 | 0.184 | 0.505 |
| connectance | 0.452 | 0.498 | 0.477 | 0.278 | 0.885 | 0.743 | 0.452 |
| NODF | 0.547 | 0.612 | 0.521 | 0.311 | 0.796 | 0.718 | 0.547 |
| MOD | -0.694 | -0.734 | -0.462 | -0.258 | -0.798 | -0.853 | -0.694 |
| skewness | -0.431 | -0.437 | 0.044 | 0.027 | -0.484 | -0.592 | -0.431 |
| kurtosis | -0.355 | -0.362 | -0.005 | 0.043 | -0.352 | -0.491 | -0.355 |
