# Supplementary material for: Robustness of rigid and adaptive networks to species loss
Source: PLoS One. 2017 Dec 7;12(12):e0189086. doi: 10.1371/journal.pone.0189086 (PMC5720727; doi:10.1371/journal.pone.0189086)
Supplement: S5 Table — (DOCX) [file pone.0189086.s006.docx]

**S5 Table:** **Variance explained measured by adjusted R^2^ from the generalised additive model fitting of robustness on specific models for different levels of robustness.**

| **Model** | **10gs** | **30gs** | **50gs** | **70gs** | **10gn** | **30gn** | **50gn** | **70gn** |
| --- | --- | --- | --- | --- | --- | --- | --- | --- |
| Modularity+  Link density+  Skewness | 0.815 | 0.827 | 0.854 | 0.671 | 0.665 | 0.684 | 0.712 | 0.699 |
| Link density+  Skewness | 0.477 | 0.443 | 0.355 | 0.333 | 0.584 | 0.612 | 0.648 | 0.698 |
| Modularity+  Skewness | 0.642 | 0.788 | 0.838 | 0.656 | 0.629 | 0.682 | 0.698 | 0.700 |
| Modularity+  Link density | 0.804 | 0.784 | 0.839 | 0.543 | 0.619 | 0.478 | 0.454 | 0.245 |
