# Supplementary material for: Robustness of rigid and adaptive networks to species loss
Source: PLoS One. 2017 Dec 7;12(12):e0189086. doi: 10.1371/journal.pone.0189086 (PMC5720727; doi:10.1371/journal.pone.0189086)
Supplement: S6 Table — (DOCX) [file pone.0189086.s007.docx]

**S6 Table: Pearson’s correlation coefficients for different variables.**

|  | Link den. | n+m | n×m | Con. | NODF | MOD | skewness | kurtosis | RC ratio |
| --- | --- | --- | --- | --- | --- | --- | --- | --- | --- |
| Link den. | 1.000 | 0.733 | 0.851 | 0.262 | 0.362 | -0.618 | -0.398 | -0.184 | 0.411 |
| n+m | 0.733 | 1.000 | 0.895 | -0.253 | -0.112 | -0.173 | 0.067 | 0.232 | 0.307 |
| n×m | 0.851 | 0.895 | 1.000 | -0.183 | -0.045 | -0.290 | -0.298 | -0.144 | 0.395 |
| Con. | 0.262 | -0.253 | -0.183 | 1.000 | 0.879 | -0.814 | -0.305 | -0.178 | 0.140 |
| NODF | 0.362 | -0.112 | -0.045 | 0.879 | 1.000 | -0.887 | -0.207 | -0.113 | -0.007 |
| MOD | -0.618 | -0.173 | -0.290 | -0.814 | -0.887 | 1.000 | 0.424 | 0.236 | -0.215 |
| skewness | -0.398 | 0.067 | -0.298 | -0.305 | -0.207 | 0.424 | 1.000 | 0.914 | -0.518 |
| kurtosis | -0.184 | 0.232 | -0.144 | -0.178 | -0.113 | 0.236 | 0.914 | 1.000 | -0.418 |
| RC ratio | 0.411 | 0.307 | 0.395 | 0.140 | -0.007 | -0.215 | -0.518 | -0.418 | 1.000 |
